# Law and biomedicine and the making of ‘genuine’ traditional medicines in global health

**DOI:** 10.1080/09581596.2019.1594696

**Published:** 2019-06-04

**Authors:** Emilie Cloatre

**Affiliations:** Kent Law School, University of Kent, Canterbury, UK

**Keywords:** Law, traditional medicine, evidence, knowledge systems

## Abstract

This paper explores the joint roles of law and biomedicine in constituting the boundary between legitimate and illegitimate (and genuine and ‘pseudo’) traditional healing. It argues that, as law and biomedicine have grown to share common understandings of the nature of knowledge, they have come to act as converging colonizing forces that displace and alter ‘other’ forms of knowing and ordering. Even as regulatory systems set out to recognize some forms of traditional medicine, they continue to operate on assumptions that disqualify knowledge, products, and actors, that do not resemble their biomedical counterparts. This leaves traditional healing systems potentially having to either operate outside the law or adapt to it by transforming themselves, potentially beyond the point of recognition, to fit better into the systems provided by law and biomedicine. The paper explores the series of dilemma this creates for those seeking to ‘regulate better’ traditional medicine.

## Introduction

This paper interrogates the convergence of biomedicine and law and its relationship to the present and futures of traditional medicine. It explores how regulation of traditional healing shapes the bound between the ‘genuine’ and the ‘pseudo’, and what this can teach us about the relationship between law and biomedicine in global health. It argues that as law and biomedicine have grown to share common understandings of the nature of knowledge and how it can be proven or attributed, they act as converging colonizing forces that displace and alter ‘other’ forms of knowing and ordering. Accordingly, even as regulatory systems set out to recognize some forms of traditional medicine, they often operate on assumptions that disqualify knowledge, products, and actors that do not resemble their biomedical counterparts. Consequently, traditional healing systems either operate outside the law, or adapt to it by transforming themselves to align to ‘legitimate’ systems of law and biomedicine. While such regulatory movements have long historical roots, they have been intensified by the advance of industrialization in biomedicine and the expansion of global markets in medicine. Throughout this paper, biomedicine is understood not only as a field of knowledge, but also as a field of practice of which marketization, industrialization and standardization have become significant constitutive parts.

Traditional medicines cannot always be proven according to the usual tests of biomedicine (Adams, 2002). As knowledge-systems, they also sit at the crossroads of legal dilemmas that contribute to the definition of the ‘genuine’ and the ‘pseudo’ in global health. Accordingly, traditional medicine is a useful entry point to question the constitution of the ‘pseudo’, understood here as that which occupies the fringes of global health, being neither fully recognized as medicine, nor entirely dismissed as ‘fake’. To be clear, I am not suggesting that the term pseudo reflects my own reading of the value of traditional medicine. Instead, I suggest that it reflects a common position in law and policy, both in global health and across national jurisdictions. Traditional medicines are bound up in questions of legality and illegality of practice, ownership of intellectual property and the politics of knowledge. In addition, traditional medicines are a particularly acute illustration of the socio-cultural and political stakes of the nexus between law and biomedicine: they fold into the long history of the emergence of biomedicine as a main actor of global health and constitute a field in which superimposed patterns of dominance and exclusion (be they colonial or gendered, for example) have been particularly acute. These features have acquired further salience through the rise of global markets, systems of manufacturing and standardization.

Since the 1970s, public health actors, notably the World Health Organization (WHO), have paid increasing attention to the potential challenges and opportunities that traditional medicines pose to public health systems (World Health Organization, 1978; WHO, 2013). Over time, policies have promoted better integration of traditional health systems with biomedicine. In response, anthropologists have been critical of such normative discourses and have argued that, in practice, such moves may facilitate the disappearance of valuable differences between traditional and biomedical systems (although one should also attend to possibilities to resist such pressure) (Janes, 1999; Lock 1990; Wahlberg, 2008). More recently, attention and policy discourses have focussed on the ‘regulation’ of traditional medicines. Regulators, encouraged by the WHO, have argued that regulation could improve the delivery of traditional healthcare (WHO, 2013). Here, the law has been tasked with finding ‘the right’ balance between protecting patients against potential misuse of traditional therapies, or, indeed, against practices or products that may be inherently dangerous, while authorizing those considered valuable or useful. Additionally, the law is deemed important in improving or maintaining standards of practice within professions, and/or for products (McHale, 2014). These ideas have been implemented with considerable diversity. However, some common features appear across regulatory systems. Notably, a seemingly neutral drive for ‘better regulation’ masks an embedded and reified set of assumptions about what constitutes genuine ‘knowledge’ from a legal perspective. For example, in its latest Traditional Medicines Strategy (2013), the WHO appears to make at least two assumptions. First, that a distinction can and should be drawn by regulation between ‘*traditional medicines of proven quality, safety and efficacy*’ (WHO, 2013, pp. 8 & 17) and their others. Although presented as an objective triage between the ‘proven’ and the unproven, the strategy also suggests that proof is constituted on a register of evidence imbued with scientific language. Second, that a legal distinction can be superimposed onto this medical demarcation to differentiate between the regime applied to therapies which can be proven or not – be it ‘mere belief’ or ‘pseudo’ therapies. Such distinctions and dichotomies have also been relevant to IP systems and the broader politics of knowledge (Coombe, 1998; Dutfield, 2009).

This paper makes two main arguments. First, that the aforementioned assumptions and their embedding in law are problematic and performative, reshaping the future of healing systems as they set out to regulate them, and effacing alternative epistemologies. Second, it argues that the role law plays in these movements, and in the constitution of the genuine and the pseudo, are not incidental to broader market forces: there is a symbiotic relationship between law and biomedicine, as joint tools of socio-economic power and governance, which reifies and obscures processes of assimilation in knowledge production. As legal systems look to regulate ‘better’ traditional medicines, they rest on the only tools that the law has developed to distinguish the genuine from the pseudo in medicine: those of biomedicine itself. The examples drawn from the paper are from a range of jurisdictions. However, the paper is primarily grounded in a project focused on the regulation of traditional medicines in Europe and Africa. I focus my analysis on the European context, while using it to reflect on other parts of the world (notably in Africa) that are seeking to, or have recently introduced, legal frameworks for traditional medicine.

## The case of the Redmeadow plant

To contextualize this paper, it is useful to illustrate some of the key patterns that have been decried in the field of traditional medicine, and the type of stories I am building this paper around. In order to facilitate this, I use a composite example of an imaginary plant – a ‘pseudo example’. Like many objects operating in the blurry space between real and fake, Redmeadow borrows its features from a gathering of realities, but these were rearranged to facilitate the arguments in this paper and to illustrate them with a ‘too-ideal-to-be-true’ (and therefore simplified) example. The value of this approach is that it distils the key features of the genuine examples, without having these entangled with details specific to those examples which are unrelated to this paper. It also enables me to point to some of the key features of the field within a single and relatively short narrative. The example of Redmeadow is inspired by both scholarly literature and everyday examples, gathered in the media and from my empirical research. For readers keen to relate it to more real-life context, I have weaved in references to both plants, healers and laws that have fed into this composite. The story illustrates the many dilemmas that plants used for medicinal purposes are caught up in when encountering the law. It is also about how such plants’ identities and possibilities are shaped by their interface (experienced or theoretical) with biomedicine. The aim of this pseudo-example is to highlight some of those dilemmas and incoherences, and their political implications. The rest of the paper turns to analysing the role that law, and its historical relationship with biomedicine, plays in such stories.

Redmeadow has been used by healers across Southern Europe and parts of Africa since at least the XVth century. It is believed to have numerous properties, notably for respiratory conditions, and as anti-spasmodic. It has also been used by midwives both around childbirth and as an arbotifacient. While Redmeadow was commonly used in Southern Europe until the nineteenth Century, it has progressively been replaced by other drugs. This is, in part, because the popular healers who transformed Redmeadow into healing decoctions have progressively disappeared from everyday health practices, to be replaced by doctors, nurses and professional midwives, and by new treatments.^1^ Furthermore, new laws in Southern European states have prohibited anyone other than pharmacists to sell Redmeadow in its unprocessed form.^2^ Used in excess, Redmeadow can have side-effects that regulators want to see controlled by pharmacists.^3^ As pharmacists lost interest in plants in Europe, and progressively stopped stocking them, Redmeadow has become rare in everyday healing in Europe. Lately, as folk herbalism is regaining interest, some would like to reopen its use and distribution, but the law limits such possibilities.

Meanwhile, in Africa, Redmeadow has followed a similar path, though complicated by colonization. While it is unclear precisely when healers started using Redmeadow, it was commonly used by the time of colonial invasion. As healers were progressively penalized by colonial powers, Redmeadow became marginalized, often hidden to avoid accusations of sorcery.^4^ Soon, colonial laws formally banned it and other plants used in rituals and healing.^5^ However, since decolonization, Redmeadow has become tolerated again. It is still not, however, fully legalized. Notably, in states where Redmeadow has been most commonly used, and even though they are usually not applied anymore, old colonial laws still only permit biomedical doctors to treat patients.^6^

Recently, however, States have started to accept that traditional healing may have value, providing it can prove its effects. How can the healing powers of Redmeadow be proven? For traditional healers, or herbalists reviving old techniques for which there is ample testimony, evidence seems aplenty. However, legally, such evidence is anecdotal, and thus insufficient. To be considered ‘proven’, Redmeadow must be studied following certain standards. This requires money, equipment and conditions that traditional healers cannot easily access, meaning it can only be proven through other means. Those with resources can provide the necessary evidence by isolating some of Redmeadow’s properties and testing them in clinical trials. However, it soon becomes clear that this evidence relates to particular chemical compounds of Redmeadow, which need to be produced in laboratory conditions. Precise measurements of how much of Redmeadow’s chemical compounds will be taken by patients are crucial for it to be legally used and sold.

Soon Redmeadow, as tested in laboratory conditions, has been replicated by various manufacturers who, with the right equipment, quickly reproduce the precise substance that has proven its efficacy. It is turned into carefully weighed tablets, packaged in foil and plastic, and distributed through global market networks. The fact that Redmeadow is a plant (even if in its new form it no longer looks likes one) which has long been used in traditional healing renders it attractive to patients around the world who are keen to use less ‘drugs’ and more ‘natural’ products. Before long, Redmeadow thrives in global markets, adapting its packaging to customers preferences.^7^ Meanwhile, for healers and herbalists, the (il)legality of Redmeadow used as a ‘plant’, and not carefully measured tablets, remains unchanged. While some continue to use it, they are severely restricted by law and their practice is undermined as potential patients are able to find the plant as tablets. Other healers decide to adapt, and benefit from the opportunities that the new faces of Redmeadow may offer. They start using Redmeadow in its new form, change their practices and use, and introduce new techniques.

In this story, the possibilities offered to Redmeadow to circulate, be used, sold, and transformed have been heavily conditioned by law. Some may argue that here the legal system has facilitated the use of a traditional plant in a form that is proven and able to satisfy the high standards of modern healthcare. However, the law has also contributed to the very redefinition of Redmeadow as a healing device: it has approved its form that is most replicable, and in turn most industrial, and more ‘like a drug’ in the sense of biomedicine. In turn, those that use or sell Redmeadow have been replaced, or changed their practices, to fit better into the biomedical system. The sets of rights and claims that surrounded Redmeadow have also inevitably changed. Those who do not fit the new system, or who do not see tablets as fitting their practice, have remained illegal. And while the system is arguably more ‘open to traditional medicine’, it may be less likely to tolerate those who do not conform. In this paper, I am interested in these various effects and what they tell us about law’s role in ordering socio-medical relationships, and about the particular relationships of law and biomedicine. A central question is what do these relationships imply for the possibilities of traditional medicines, such as Redmeadow, to be considered as ‘genuine’ objects of healthcare? If we are to facilitate the use of ‘traditional’ healing, could the law be reimagined in a way that enables Redmeadow to be seen as a healing device in and of itself?

## Approaching law in global health

In thinking through this issue, I approach law as a complex, often unpredictable, but also politically charged arbiter of ‘genuine medicine’. We know that law participates in setting the boundaries of legitimacy, and indeed often works jointly with science to produce these boundaries (Cloatre & Pickersgill, 2014). Socio-legal scholarship has also shown that law is contingent, uncertain, and multidirectional, resulting in modes of action that are not always (or not only) those it claims to follow, and resting on socially and politically loaded assumptions, and histories, that it does not always make apparent. Finally, law is performative, producing some of the realities that it sets out to regulate (Cloatre & Cowan, 2019).

Those features of law are particularly interesting in the context of medicine, and examining the interactions between these two powerful institutions renders visible some of the patterns of power that they produce, both individually and together. Law, like biomedicine, claims particular forms of neutrality and objectivity; yet, like biomedicine, it has a long history of violence and exclusion. Global health, understood here as the gathering of spaces in which global inequalities in healthcare are formed and challenged, provides important sites and perspectives from which to reflect on law and biomedicine. Such features play out at different levels, and through multiple legal and regulatory mechanisms, in the context of traditional medicine. Such legal mechanisms includes those pertaining to the regulation of products (including, as a core example, herbal medicines), both in relation to their marketing (eg. market authorization, traditional herbal registration) and to the place and conditions of their sale (prescription vs over the counter medicines; pharmacists vs herbalists vs supermarkets). Such regulations, in addition to formal state requirements are also created by soft law, guidelines, codes, and standardized documents. Other relevant regulations include those on therapeutic practices (including the civil law concept of ‘illegal medical practice’, later exported to colonies); professional regulations and registrations; or rules on training and protections of title. Jurisdictions vary in how those connect and are organized. While some systems have preserved significant monopolies of therapeutic practice for biomedical professionals, others have introduced a selective recognition and regulation of specific traditional medical systems. Finally, some states have sought to organize in a generic manner ‘traditional medicine’ and often focus implicitly on legitimizing and valuing local traditions of healing.^8^

In the remainder of this paper, I argue that although some of these approaches to law seem a priori more appealing than others to those who want to promote and value traditional medicine, relying on law in itself carries a certain political and ideological ‘baggage’. This means that the role of law as one technique of legitimation needs to be observed critically, both for its effects on the regulated field, and for practices that fall outside of it. Of course, law is only one mode of legitimation for traditional medicine: however, given its keen embracing by the WHO and others, it seems useful to put it to scrutiny.

My examination of traditional medicines builds on two strands of legal scholarship. One examines illegality, and argues that illegality is best conceived as a state of precarity and negotiation (Cloatre, 2018; Cloatre & Enright, 2017; Peterson, 2014). Boundaries between legality and illegality are always revisited and transgressed, producing ‘grey’ or ‘pseudo’ zones of careful practice and negotiation rather than the clear dichotomies that regulators may set to create. When thinking about the effects of law and illegality in practice, it is important to think not only about the direct, planned or expected effects of law, but also about its effects in reshaping social relations in excess of its stated aims. Here, the question is about exploring what legal restrictions mean for the everyday uses of plants like Redmeadow and associated practices. The other strand of legal scholarship examines ownership and valuing in intellectual property law (IP). One effect of IP is to allocate value to the ‘genuine’ against intrusions from the ‘fake’ or ‘pseudo’. As others have demonstrated, IP regimes are deeply political, and representative of particular Eurocentric and industry-driven understandings of what constitutes ‘genuineness’ yet they mediate the possibilities for particular knowledges and products to access markets and condition their value (Coombe, 1998; Krikorian & Kapczynski, 2010). Straddling these two sets of questions, traditional medicines are a unique site from which to question the divide between practices considered sufficiently valuable and real to be integrated within public health systems, and those permanently excluded.

## Power relations through law and biomedicine

As legal systems seek to determine what can lawfully be sold or practised, questions arise as to what kind of knowledge can help arbitrate the boundary between the ‘genuine’ and ‘pseudo’. In this section, I argue that assumptions by the WHO and others that ‘proven and genuine’ traditional medicines can be separated from the unproven and/or illegitimate derive partly from the dominance and colonizing nature of biomedicine and from its long-standing embracing by the law. This has been rendered more salient by the embedding of biomedicine in industrial modes of production and the demands of global markets. Law and biomedicine have, arguably, shared and deployed a narrative about the nature of genuine knowledge in medicine since at least the nineteenth century: that genuine, modern knowledge should be aligned with a particular scientific discourse, provable through scientific tests, and that it should be clearly separated from spirituality and ‘beliefs’ (Adams 2002; Gaudillière, 2007). These assumptions remain present, even where policies suggest a need to more generously embrace the ‘others’ of biomedicine. To understand the implications of such assumptions, it is useful to briefly look first at their histories, even if for reasons of space these can only be sketched. While the colonizing history of biomedicine and law have been thoroughly explored individually, their interaction has been given less specific attention.

### European context

In looking at law’s role in facilitating the expansion of biomedicine within Europe and colonial contexts, a first (perhaps obvious) point to make is that this role was not confined to legitimizing ‘truth’ or ‘scientific evidence’, but was intimately connected to broader political and institutional struggles for empowerment and disempowerment (Porter, 1999; Ramsey, 1977). In Europe, the driving away of other healing practices by law (including criminal law), as law embraced the scientific logics of biomedicine, were also about the reorganizing of powers such as those of church vs state, popular culture vs the elite, and the role of women in public life (Ehrenreich & English, 2010; Ramsey 1999). In the various movements that surrounded the legitimizing and delegitimizing of various professionals, with and through law, and as the make-up of the sphere of ‘genuine healthcare’ was redesigned, processes of marginalization at play were linked to broader trends of power and exclusion. The role of law in excluding forms of medical knowledge along lines that met those of class or gender was sometimes direct (for example in forbidding particular practices, or earlier in the definition of what constituted witchcraft rather than healing), and sometimes indirect (for example, in France, as biomedicine became the only legally recognized means to treat patients, women were not admitted in universities – and therefore effectively denied the possibility to be legal healers). In both cases, the result was the organizing of lines of legitimacy in care and medicine along lines of dominance and exclusion that were political; their embedding in scientific narratives, however, disguised them as otherwise.

### Traditional healing and colonialism

With colonial expansion, and as biomedicine became a powerful tool of domination and population control, the separation of ‘knowledge’ and ‘belief’, or ‘rational’ and ‘irrational’, was central to the settlement of socio-political power (Bigon, 2012; Echenberg, 2001; Langwick, 2011; Vaughan, 1991). Here it is worth recalling two key aspects of the history of colonial medicine. First, that legal rationalities played a significant role in the settlement of power through knowledge that underlies much of this history (Comaroff, 1993; Echenberg, 2001). Throughout colonial history, the claims of biomedicine as a particular source of objective knowledge became embedded and solidified in regulatory decisions (for example, in determining who was legally allowed to be in certain spaces, where housing and settlements could be built, but also which healing practices were permissible). Arguing that recently acquired (yet often uncertain) biomedical knowledge was ‘more useful’ (or ‘more modern’) than its local alternatives, legal institutions set to reorganize populations in a way that violently empowered some over others.

Second, colonial medicine worked to alter and replace local nosologies and healing traditions with its own classifications and definitions (Langwick, 2011). In organizing governance over bodies and populations, lines were redrawn between what could be seen as constituting healing or witchcraft, knowledge or belief. These lines operated both as specific sites of interventions in medical practices, and as part of a broader process of controlling through knowing and unknowing. This broader process was, in turn, at times animated by an ambivalent effort to borrow yet reject, or to appropriate and assimilate. These dynamics are still pertinent today and reflected in the ambivalence underlying the regulation of traditional medicine, which I return to below (Adams, 2002; Wahlberg, 2008).

### Industrialization and the demands of global markets

The joint history of law and medicine became complicated further by the relationship between regulation, biomedicine, and industrial modes of production. As biomedicine became an industrial enterprise, it also became dependent on new systems of authorization, standardization and replicability. Measurements, procedures and devices became necessary to be able to prove that a drug released to circulate in the market was precisely that which had been authorized (Gaudillière, 2013). The very nature of biomedicine as an institution became co-dependent on those modes of organization and validation (Gaudillière, 2007; Keel, 2011). The need to demonstrate ‘efficacy’ became an integral part of this institution: for medicines to be approved and considered as such onto markets and in law, it became inevitable that they should be able to demonstrate their efficacy, and an efficacy that could be replicated under any similar circumstance, and in which subjectivity, faith and personal beliefs could have no place.

The ambiguity in how the WHO approaches the division between ‘knowledge’ and ‘belief’ can be read in the light of these particular histories. If the WHO accepts that beliefs may be recognized as part of traditional medicine, the place of such practices in the health system is also denied by the need to regulate through proof of ‘efficacy’. Elsewhere in WHO guidelines and documents, this has been translated into a need to fit into particular procedural and standardized models that are characteristic of biomedicine, and seek to isolate objective physical and measurable phenomena from individual experience. Where beliefs, faith, or the ‘unexplained’ form a constitutive part of health practices, some of their elements may sit outside such remit of proof, and their efficacy may become unprovable to those seeking a purely objective or physical demonstration (see also Friesen, 2019). The question of what to regulate, and how to regulate it, cannot exclusively be solved by an attention to therapies that have been able to prove themselves under the terms of biomedicine. This however creates dilemmas for regulators that I turn to in the next section.

## Law, biomedicine and the displacement of alternative epistemologies in healing

Redmeadow’s dilemmas can be explored through a critical evaluation of how law and biomedical expectations continue to shape possibilities for genuine alternatives in health. In this last section, I argue that efforts to distinguish ‘scientifically proven’ traditional medicines from ‘beliefs’ may contribute to further reifying the limits to pluralism that law and biomedicine have invested in creating and maintaining. This can be seen through two movements: first, the continuing exclusion and precarization of practices that are not ‘provable’ under the terms of biomedicine, where regulatory systems embrace such understanding of proof uncritically; second, a process of assimilation, where alternative practices are incorporated into formal health-care systems only if they ‘look more like’ biomedicine. I explore each of these effects in turn. Readers should note that I leave out one scenario here, where traditional medicine would be neither legal nor illegal but left out of law altogether and regulated through alternative systems of ethics or traditional practice – where medical pluralism meets legal pluralism. Here, my focus is instead specifically on what centralized state regulation means for traditional medicine, and why it deserves careful and critical attention.

### Law, evidence and their ‘misfits’

In this section, I explore the implications of failing the standards set by law to determine what constitutes ‘valid’ traditional medicine in the eyes of the state. Although each legal system will design those boundaries with varying rigidity, some practices will always overspill and fail to meet regulatory standards of proof. In some legal systems, practices that have not proven themselves sufficiently or through the right routes might simply be ‘unregulated’, and therefore permitted unless explicitly breaking the law (as is arguably the case in the UK). Their position ‘outside’ the law will impact, however, on their ability to thrive: for example, they will be limited to the private spaces of healthcare, and titles will not be protected by the state. More striking however are jurisdictions where therapies and therapists that are not explicitly regulated are ‘illegal’, as in France. Notably, where new systems of regulation are put into place to recognize the ‘proven’, and depending on the phrasing of the law, therapies that have not been proven may de facto be brought into illegality.

However, as both socio-legal scholarship and everyday events demonstrate, illegal practices never disappear: they instead become hidden from view, which often results in a certain precarity of practice and use (Cloatre & Enright, 2017). ‘Illegal healing’ is no exception here. For instance, in France, despite very strict laws restricting all ‘diagnosis and treatment’ to biomedical doctors and no legal recognition of professions such as naturopaths or Chinese healers, alternative medicines are known to operate (Cloatre, 2018b). In such contexts, unlawful practices tend to develop careful systems of negotiation to ensure that they remain within certain lines of acceptability: this may mean remaining hidden away from public view, or operating within particular lines of representation, rhetoric or practice, and it always means organizing to avoid crossing the (often unpredictable) lines that may bring formal sanctions (Langwick, 2011; Marsland, 2008). Effectively, for the law, these practices come to constitute the spaces of pseudo-healing, outside what regulatory regimes formally recognize. Importantly, the precarity that this potential illegality may produce has safety implications: ‘the pseudo’, once implicitly stamped as such by law, is less formally governed and therefore less ‘safety-checked’. The heavy systems of checks and regulations that surround ‘health professions’ and ‘medicinal products’ are not often applied to the same extent to treatments and individuals excluded from these categories. Here, the very aim of regulation to facilitate better health through better safety may be more difficult to obtain if it does not account for the possibility to engage the pseudo as well as the proven.

Furthermore, those that are legally unproven and do not fit the law’s understanding of knowledge also face precarity in ownership and valuing. The misfit of global intellectual property with traditional knowledge systems has been extensively documented (Coombe, 1998). In the eyes of intellectual property law, valuable knowledge continues to be, predominantly, a particular form of processed knowledge defined under the terms of science. This idea of value has implications for the genuine and the pseudos of global markets: as they are stuck between precarity of practice and precarity of valuing, pseudo-medical systems are also inevitably confined to the edges of global markets (Adams, 2002). Products that can travel are also those that get financially rewarded because they are identifiable at a particular point in time, traceable to a particular process of invention, and replicable in ways compatible with industrial production and are thus ‘genuine’ in the eyes of the law. Those able to engage most with tests and procedures designed for biomedicine can be both legitimate actors of health provisions and legitimate knowledge-holders. Law is important here because the global markets which therapeutic products, or indeed healers, may seek to access are shaped by such regulatory possibilities.

### Regulation, displacements and assimilation

Having looked at the double precarity experienced by ‘unproven’ products and practitioners, this section considers what happens to therapies that are deemed ‘genuine’. It focuses on the processes of ‘inclusive exclusion’ that are at play in the transformations, negotiations and adjustments required to be deemed ‘genuine’. As the law sets out to regulate ‘better’ the messy field of traditional medicine, it contributes to reshaping the very definition and nature of that field, often in excess of the law’s stated aims. This happens through a process of negotiation and assimilation, in which responses to legal regimes reinforce the influence and epistemologies of biomedicine. If legal systems are to rest on a division between that which can be scientifically proven and that which cannot an effect is to trigger other therapeutic knowledge-systems into explicitly positioning themselves in relation to science (notably in its industrial expression) as they seek to engage with law.

An important characteristic of industrial science, relevant both in the transmission of knowledge, but even more visibly to the making of products, is its replicability. ‘Proven’ therapies are also those that are able to be perfect duplicates of what has been tested. For products, escaping the sphere of the pseudo is therefore not only about being proven, but also dependent on both an ability to be measured and to be replicated (Ecks, 2013; Wahlberg, 2008; Osseo-Asare 2014). This ability often depends on the product itself: plants are inherently less measurable than their industrially produced extracts, which in turn renders raw herbal treatments less replicable than manufactured ‘herbal products’ – Readmeadow itself is less replicable than its tablets.

Similarly, where professions seek further recognition by the law, they commonly seek to enrol the framings of science that the law expects. States may also require forms of training and knowledge transmission for its traditional healers that are akin to science. For example, herbalists in France seek to engage pharmacology in their quest for formal recognition (Cloatre, 2018b; Garreta, 2006), and courses in traditional medicines are part of recent regulatory framing of traditional medicine in Ghana (Cloatre, 2013). Such engagements with science by the professions are always done with some ambivalence. Tradition and science may be called upon selectively to assert both difference from the biomedical, and assimilation of the discourses and modes of recognition that surround it. As healers seeking recognition navigate between efforts to be recognized through assimilation and through difference, assimilation is crucial to being legally accepted, although there is always scope to creatively resist the law and its preferred model of practice (Pordié & Hardon, 2015). Overall, as regulators seek to engage more actively alternative or traditional medicines, they are also expecting them to be more standardized, replicable and auditable, both in terms of their professions and of their products (Bivins, 2010; Wahlberg, 2008). Those who do not comply will remain in the grey zones of unlawfulness.

## Conclusion

As therapies are folded into legality, or are required, through law, to be proven and replicable, an effect of regulation is the transformation of the products and practices in question. Of course, at one level, this is precisely the aim of law: for example, to promote safety, regulation may mean that professional qualifications and product quality will be checked; or indeed that the line between what ‘works’ and what does not need to be drawn. However, the law always has effects that do not form part of its official script: it is always performative, creating the realities it has imagined but also producing unintended consequences.

In the field of traditional medicine, the lines between the genuine and the pseudo are navigated and established through regulation. While contemporary regulation seemingly embraces a broader range of therapies, regulation also contributes to making the ‘genuine’ more uniform, and, in effect, more like biomedicine. Such transformations of therapeutic practices in response to regulation are visible around the globe, and are about more than merely adjusting to be more ‘performant’ – or in other words about more than creating ‘better therapies through better regulation’: they deeply transform and translate the very nature of particular therapies (and, through them, networks of empowerment in medicine). Legally recognized practices may bear little resemblance to the systems and ideologies that they originated from, while those continue to operate on the edge of official legal systems. Redmeadow becomes Redmeadow-pills on global markets, remaining as Redmeadow plant mostly in discreet illegal spaces – and in nature, of course.

Importantly, as the drive towards proof and replicability separates the proven from the pseudo, and knowledge from belief, the spiritual dimension of particular healing systems disappears from sight, transforming the cultural make-up of those therapies: it is the ‘thing’, and not beliefs surrounding it, that are at stake when dealing with proof. Here, the law participates in reshaping other systems to appear less like alternatives, and more like biomedicine. As law has come to understand the boundaries of legitimacy through biomedical language and logics, it is necessary to question to what extent regulating diversity without restraining such diversity, and regulating without assimilating, remains possible. Importantly, these processes also create new lines within: as the ‘genuine’ is expanded in scope, yet assimilated in its making and practice, the unassimilated becomes ‘othered’. Within particular practices, strands that are legitimated and those that are not, may split and co-exist, generating new spaces of ‘genuine’ and ‘pseudo’ therapies, and legality and illegality: healers wishing to continue using Redmeadow as a plant will do this in the precarity of illegal spaces, while those willing to turn to pills will have new opportunities. Whether law can be turned to without triggering such processes remains an open question.

The point here is not to deny the value of state regulation of traditional medicine per se. Indeed, within the demands of regulation and global markets, some Asian medicines in particular have successfully developed new paradigms and what Pordié and Guaudillière (2014) have analysed as an ‘alternative modernity’ in the pharmaceutical making. However, such capacity to resist, deviate and reinvent is not always successful, and requires significant institutional and economic power. For regulators keen to reinvent the traditional in a new form of modernity, there is value in paying attention to ‘what happens next’ when new legal regimes are created, and to anticipate the displacements at play: notably, zones that fall into grey areas of regulation and practice need to be anticipated and accounted for. For some practices, legitimacy may depend neither on the terms of biomedicine nor those of the law, and it may be resistant to external intervention or superimposed categories of validity.

## Notes

1. Including under the effects of new legal restrictions, such as notably in France with the Loi du 19 Ventôse an XI (10 March 1803) on illegal medical practice.

2. For example in France only pharmacists are allowed to sell plants (bar an list of 148 exceptions – on which the plants cited in fn12 do not belong) since 1941.

3. Here and later in the story, readers will find features of Redmeadow echoing with the stories of numerous plants such as ephedra, calendula, cornflower, artesimia, or St John’s wort.

4. For an exploration of the complex interface between witchcraft, healing and regulation see Ashfordth (2005).

5. Here the story simplifies some of the multiplicity in the application of colonial laws traditional healers. The transfer of the1803 law on illegal medical practice by France to its African colonies, where it was applied in a variety of ways, is a notable example.

6. For example, this is the case in Senegal or Cameroon, although it is not straightforwardly applied in practice.

7. As a comparison see the story of Hoodia as recounted in Foster (2016).

8. Here one may think of the examples of France or Senegal for the first scenario (though with contrasting stakes and practices); China, India or Mauritius for the second; and Ghana or Burkina Faso for the third.
